# “Translational” Patient Education in Adult Cancer Patients with Entero-Urostomy and Their Caregivers: A Qualitative Focus Group Study

**DOI:** 10.3390/healthcare14162501

**Published:** 2026-08-12

**Authors:** Nicolò Panattoni, Alessia Campoli, Alessandro Spano, Carmelina De Stefano, Daniele De Rossi, Federica Sorrenti, Albina Paterniani, Aurora De Leo, Fabrizio Petrone, Laura Iacorossi, Emanuele Di Simone

**Affiliations:** 1 Nursing Research Unit IFO, IRCCS Regina Elena National Cancer Institute, 00144 Rome, Italy; nicolo.panattoni@ifo.it (N.P.); alessia.campoli@ifo.it (A.C.); carmelina.destefano@ifo.it (C.D.S.); federica.sorrenti@ifo.it (F.S.); aurora.deleo@ifo.it (A.D.L.); fabrizio.petrone@ifo.it (F.P.); 2 Department of Biomedicine and Prevention, University of Rome Tor Vergata, 00133 Rome, Italy; 3 School of Nursing, Sapienza University, IRCCS Regina Elena National Cancer Institute, 00144 Rome, Italy; derossi.2138372@studenti.uniroma1.it (D.D.R.); albina.paterniani@ifo.it (A.P.); 4 Department of Life, Health and Health Professions Sciences, Link Campus University, 00165 Rome, Italy; l.iacorossi@unilink.it; 5 Department of Medical, Movement and Wellbeing Sciences, Parthenope University of Naples, 80133 Naples, Italy; emanuele.disimone@uniparthenope.it

**Keywords:** patient education, cancer patient, caregiver, ostomy patient, entero-urostomy, focus group, qualitative study

## Abstract

**Highlights:**

**What are the main findings?**

- The study identifies translational patient education as a novel, dynamic process integrating clinical, peer, and relational knowledge through interactions between patients, caregivers, and nurses.
- Patient education delivered within a focus group setting fosters peer support, enhances experiential learning, and supports psychological and practical adaptation to life with a stoma.

**What are the implications of the main findings?**

- Patient education programmes should be designed as interactive, person-centred processes that actively involve patients and caregivers and incorporate peer-based approaches.
- The development of structured frameworks and targeted training for healthcare professionals is needed to implement translational patient education across diverse clinical settings.

**Abstract:**

**Background/Objectives**: Patient education (PE) is a key component in the management of chronic conditions, including living with an entero-urostomy. Exploring patients’ and caregivers’ experiences may inform the development of tailored educational interventions. This study aimed to explore the perceptions, experiences, and needs of adult oncology patients with entero-urostomies and their caregivers as part of the patient education process. **Methods**: A descriptive qualitative study was conducted at a single Italian cancer centre using two focus group sessions integrated with PE activities. Discussions were audio-recorded and analysed through thematic analysis following the framework described by Dawadi. NVivo was used to support data management and facilitate the assessment of data saturation. **Results**: A total of 28 participants (15 patients and 13 caregivers) were recruited. Three main themes emerged: translational patient education as a co-constructed educational process, time as a protective and regulatory resource in everyday stoma management and living with a stoma requires adaptation and meaning-making. The first theme describes a dynamic integration of clinical, peer, and relational knowledge co-constructed by nurses, patients, and caregivers. The second theme referred to the perception of time and care contexts, including telenursing, as protective spaces supporting anticipatory coping and daily life planning. The last theme captured the embodied and psychological adaptation to living with a stoma, characterised by heterogeneous processes of acceptance and meaning-making. **Conclusions**: PE emerges as a relational and interactive process that supports self-care, emotional adjustment, and quality of life. The concept of translational patient education, as part of the patient education process, offers a novel framework for integrating multiple forms of knowledge within educational practices, strengthening the person-centred nature of PE programmes. These findings support the development of tailored, person-centred PE interventions that incorporate the dynamic educational ecosystem involving patients, caregivers, and stoma care nurses. They also highlight the need for further research to validate this conceptual model across diverse settings and populations.

## 1. Introduction

Patient education (PE) is conceptualised as a range of structured educational interventions delivered by trained health professionals to support patients living with chronic conditions in enhancing their self-management capabilities, with the active involvement of carers and family members [1,2]. PE plays a crucial role in empowering patients with the appropriate knowledge, skills, and understanding of disease processes, enabling them to manage their condition effectively in collaboration with healthcare professionals [1]. Nurses are central to this process, supporting patients’ cognitive appraisal of the disease and its symptoms through educational and supportive interventions [3,4,5]. Evidence shows that PE interventions, delivered through a wide range of strategies and technologies, improve biomedical outcomes, psychological well-being and psychosocial functioning in individuals with chronic conditions [1,6].

In the literature, patients living with an ostomy are often conceptualised as living with a chronic illness [7,8]. An ostomy is a surgically created opening on the abdomen that enables the external diversion of faecal or urinary output [9]. The predominant indications for ostomy creation are bladder cancer, inflammatory bowel disease and colorectal cancer; the corresponding types of stoma created are urostomy, ileostomy, and/or colostomy, respectively [10].

Epidemiological data highlight the growing relevance of these conditions: in 2024 the Italian Association of Cancer Registries (AIRTUM) reported 48,706 new diagnoses of colorectal cancer (27,473 men and 21,233 women) and 31,016 new diagnoses of bladder cancer (25,227 men and 5789 women) [11,12].

Ostomy creation may affect individuals across multiple dimensions, including physical, psychological, social, and spiritual aspects [13]. Limitations in physical functioning, dietary restrictions and sexual difficulties are among the key factors influencing quality of life [14]. In oncology, cancer stage influences concerns related to the stoma and social and family relationships [15].

PE is recognised as a core component of nursing care throughout the entire care pathway of patients living with a stoma [16]. Preoperative assessment is essential for psychological preparation, while postoperative interventions have been shown to significantly improve self-efficacy and health-related quality of life (HRQoL) in individuals living with a urostomy [10].

Peer-delivered educational interventions also contribute to improved knowledge, attitudes, and practices and enhance the overall quality of life in individuals with permanent ostomies [17]. Focus groups represent a qualitative research method in which a trained moderator guides a structured discussion among participants to explore shared experiences related to a specific phenomenon [18]. This interactive format encourages participants to respond to one another, compare experiences, and collectively construct meanings. Moreover, the phenomenon described by Morgan as the lightning strike represents a recognised feature of focus group discussions, whereby the introduction of a topic by one participant spontaneously stimulates contributions from others and shapes the direction of the discussion without requiring direct moderation by the facilitator [19].

Exploring the perceptions and experiences of patients with enterostomies or urostomies, as well as those of their caregivers, can support the tailoring of educational content and care interventions to their specific needs. Recognising the value of interaction and shared discussion among patients, we selected a focus group approach for our study. Previous studies have explored the experiences of ostomy care dyads, focusing on psychosocial adaptation and caregiving relationships [20]. To our knowledge, no previous studies have investigated the educational experiences and perceptions of adult cancer patients living with an enterostomy or urostomy and their caregivers by exploring both groups simultaneously within the same qualitative inquiry, underscoring the need for research that captures the relational and experiential dimensions of patient education in this population. This study aimed to explore in depth the experiences, perceptions, and needs of adult cancer patients with entero-urostomies and their caregivers in the context of PE.

## 2. Materials and Methods

### 2.1. Study Design

A single-centre descriptive qualitative study was conducted using focus group discussions [21], with data analysed through a thematic analysis approach [22] to identify and organise emerging themes. The study was implemented at the IRCCS Regina Elena National Cancer Institute (Rome, Italy) in May 2025. The COREQ Guidelines (Consolidated Criteria for Reporting Qualitative Research) were used to improve the study’s consistency, quality, and rigour (Table S1) [23].

### 2.2. Population and Setting

The study sample included adult cancer patients with an enterostomy and/or urostomy and their caregivers, recruited using a consecutive sampling technique, representing a group relevant to the research question [24], from the Stoma Care Nursing Outpatient Clinic for Ostomy Management at the Regina Elena National Cancer Institute, Rome, Italy. Patients were enrolled in the study according to the following inclusion criteria: (1) ≥18 years, (2) adult oncology patients with an enterostomy and/or urostomy, (3) patients followed by the Stoma Care Nursing Outpatient Clinic for Ostomy Management at the Regina Elena National Cancer Institute, Rome, Italy, (4) patients willing and able to participate in the focus group discussion; (5) patients able to understand and speak Italian and to provide informed consent for study participation and data processing, either directly or via a designated witness. Patients were excluded from the study if they met one or more of the following criteria: (1) unable to actively participate in the focus group and/or educational activities; (2) lack of autonomy in adhering to the procedures required by the study. Caregivers meeting the following inclusion criteria were included in the study: ≥18 years, caregivers of adult oncology patients with an enterostomy and/or urostomy followed by the Stoma Care Nursing Outpatient Clinic for Ostomy Management at the Regina Elena National Cancer Institute, Rome, Italy, caregivers available and able to participate in the focus group discussion; caregivers able to understand and speak Italian and to provide informed consent for study participation and data processing, either directly or through a designated witness. Caregivers were excluded if they were unable to actively participate in the focus group and/or educational activities or lacked autonomy in adhering to the procedures required by the study. The aims and procedures of the study were illustrated to the patients prior to signing the informed consent form and the commencement of the focus group.

The estimated sample [25] comprised 14 patients and 14 caregivers, divided into two focus group sessions with approximately seven patients and seven caregivers in each session. Participants were recruited until data saturation [26] was achieved, defined as the point at which no new themes or meaningful insights emerged from the focus group discussions. Data saturation was reached with a final sample of 15 patients and 13 caregivers. No eligible participants refused to participate. Focus groups are designed to generate in-depth data from purposively selected individuals, typically ranging from four to fifteen participants, rather than from a statistically representative sample of a larger population [21].

### 2.3. Patient Education Methodology

Two PE sessions, each integrated with a focus group discussion, were conducted. The focus group duration ranged from 2 to 3 h. Patient education was delivered concurrently with the discussion and tailored to participants’ responses to each guiding question. The sessions were led by a head nurse (A.S., RN, MSN) who had received specific training in PE [27]. The moderator had no prior relationship with the participants and no professional background in stoma care.

Participants’ privacy was ensured: the focus group was conducted in a private and confidential setting. In accordance with the Patient Education Guidelines [2,28], PE interventions addressed different domains depending on the needs expressed during the discussion: theoretical knowledge of the stoma (cognitive learning domain, including knowledge, concepts, procedures, and principles); practical skills (gestural learning domain, related to technical and manual abilities); and socio-relational aspects (relational learning domain, concerning social, family, and occupational relationships).

### 2.4. Data Collection and Assessment Tools

All participants completed a purpose-designed socio-demographic and clinical information questionnaire. Socio-demographic variables included age, sex, level of education, marital status, employment status, and household composition. Clinical variables comprised diagnosis, date of diagnosis, previous treatments, and current therapy.

The focus group was conducted by a nurse experienced in this methodology and trained in PE. To facilitate discussion among participants, a set of prompting questions was used: “*(1) Can you describe how you felt when you found out that you would have to undergo surgery for the creation of a stoma (and/or when the person you care for was informed that they would have to undergo such surgery)?; (2) What were the main changes or difficulties you experienced in your daily life following the creation of the stoma?; (3) Can you describe your experience with the stoma care nurses during outpatient nursing consultations?; (4) What does receiving education on enterostomy management mean to you?; (5) Was there anything that prevented you from following the recommendations or instructions you received?; and (6) If you could modify the educational programme throughout the care pathway, what changes would you suggest?*”.

At the conclusion of the focus group session, participants completed a satisfaction questionnaire based on a five-point Likert scale to evaluate PE activities.

The focus group was digitally audio-recorded to facilitate the qualitative analysis of the collected data.

### 2.5. Data Analysis and Trustworthiness

Data analysis procedures were performed by two researchers with expertise in stoma care (A.C., RN, MSN, PhDc; N.P., RN, MSN, PhD). A third researcher with expertise in qualitative research methodology (L.I., RN, MSN, PhD) was involved in resolving discrepancies through discussion until unanimous agreement was reached. The multidisciplinary research team comprised nurse researchers with expertise in qualitative methodology and stoma care, whose different professional backgrounds contributed to the interpretation of the findings. Throughout the analytical process, the researchers engaged in reflexive discussions to critically consider how their clinical experience, prior knowledge, and assumptions could influence data interpretation. Verbatim transcripts of the digitally audio-recorded focus group were elaborated and grouped based on the guiding question. The analysis was based on verbatim transcripts of the focus group discussions. No field notes or systematic observations of non-verbal behaviours were incorporated into the thematic analysis, as the study aimed to identify themes emerging from participants’ verbal accounts. The focus group discussions were analysed using a thematic analysis approach in accordance with the six phases proposed by Dawadi (2020): (1) familiarisation with data; (2) generating initial codes; (3) searching for themes; (4) reviewing themes; (5) defining and naming themes; (6) writing the report [22]. NVivo Version 14.0 was used to facilitate qualitative data management by supporting systematic coding, theme development, and the evaluation of data saturation. Member checking was not included in the original study protocol and was therefore not undertaken.

The criteria proposed by Lincoln and Guba [29] were adopted to ensure the trustworthiness of the study, specifically credibility, dependability, confirmability and transferability. To ensure dependability, triangulation was employed, involving the participation of two or more researchers during the analysis in order to reach agreement from different perspectives, thereby enhancing the breadth of the phenomenon under investigation and allowing for multiple interpretations. Confirmability was ensured through the use of audit trails, whereby two researchers who were not involved in data collection or analysis independently reviewed the research and analytical processes to verify the consistency of the findings. Finally, transferability was ensured through a clear and detailed description of the methodology, research process, and results. Credibility was supported through the systematic organisation of data and the transparent documentation of analytical decisions throughout the process.

### 2.6. Ethical Considerations

The study was conducted in accordance with the Declaration of Helsinki [30], and Good Clinical Practice (GCP) guidelines and applicable regulations, ensuring the protection of personal data in compliance with Regulation (EU) 2016/679 and national data protection laws. The study was approved by the Territorial Ethics Committee Lazio Area 5 of IRCCS Regina Elena National Cancer Institute—IFO (Protocol Code No. 2138, 11 February 2025; Experimental Registry No. 290/IRE/24). All participants provided written informed consent before enrolment. Participation was voluntary and without financial compensation, and individuals were informed of their right to withdraw from the study at any time without any consequences for their clinical care pathway. Confidentiality and anonymity were strictly ensured throughout the study. Data were coded and analysed in aggregate form to prevent participant identification, and the transcription and analysis processes were conducted in a manner that precluded traceability to individual participants. All nurses involved in the study completed a PE training programme before study implementation [27].

## 3. Results

### 3.1. Socio-Demographic and Clinical Participant Characteristics

A total of 28 participants took part in the study, comprising 15 oncology patients living with an enterostomy and/or urostomy and 13 caregivers, all of whom were the patients’ partners. The sociodemographic and clinical characteristics are shown in Table 1. The sample was mainly composed of predominantly male patients and predominantly female caregivers. A total of 80% of patients and 92.3% of caregivers reported being married or cohabiting. The majority of the sample had a colostomy (46.7%), and one patient had both a colostomy and a nephrostomy. The largest proportion of participants (33.3%) had been living with a stoma for three years at the time of the focus groups.

High levels of satisfaction were reported at the conclusion of the focus group, with mean scores close to the maximum value of 5 (4.8 for both patients and caregivers), indicating a very positive evaluation of the experience.

### 3.2. Focus Group Findings

The analysis of the focus groups identified three emerging themes. The first theme included three subthemes, while the remaining two themes each comprised two subthemes. The themes were: translational patient education as a co-constructed educational process, time as a protective and regulatory resource in everyday stoma management, and living with a stoma requires adaptation and meaning-making. Table 2 presents the detailed qualitative findings.

We provide an overview of the themes and subthemes supported by excerpts from the focus group verbatim (reported in italics), indicating whether the statement was stated by the patient [Patient] or by the caregiver [Caregiver].

#### 3.2.1. Translational Patient Education as a Co-Constructed Educational Process

The experiential knowledge of patients represents an educational resource that has not yet been fully formalised. Knowledge translation in healthcare does not occur solely through professionals transmitting scientific evidence to patients. Rather, patients themselves transform lived experience into shareable knowledge. In this perspective, patient education cannot be understood as a linear process of information transfer, but as a circular and reciprocal exchange of knowledge. Within this dynamic, patient education assumes a translational dimension, wherein experiential knowledge and scientific knowledge interact to generate what may, to our knowledge, be described for the first time as translational patient education, an educational ecosystem in which the stoma care nurse functions not only as the provider of therapeutic education but also as the facilitator and mediator of knowledge exchange between patients and caregivers. In turn, patients and caregivers are not merely recipients of education but active contributors who transform lived experience into transferable knowledge that informs other patients, caregivers, and, potentially, healthcare professionals. In this context, the term translational refers to the multidirectional circulation, mediation, and co-construction of knowledge across all actors involved, rather than to a new educational intervention. Three forms of literacy emerge from this process: clinical literacy, peer literacy, and relational literacy.

##### Ostomy Specialist Literacy

Most participants describe the educational support provided by stoma care nurses as a cornerstone of their adaptation process. Through clinical expertise, practical demonstrations, and emotional reassurance, nurses offered a form of specialist literacy that helped patients and caregivers manage the stoma in everyday life. This represents a form of clinical literacy (ostomy specialist literacy), delivered by stoma care nurses through specialist knowledge and clinical expertise and grounded in the framework of therapeutic education.

All participants expressed a strong sense of support and reassurance provided by stoma care nurses.

*Guardian angels […] you come to support us, and we rely on that a great deal. It makes us feel that someone has our back*

*[Patient]*

*Stoma care nurses helped me when I had difficulties. When I was still inexperienced and noticed redness or burning, they advised me on the treatment and how to deal with it*

*[Patient]*

Several patients and caregivers described their interactions with stoma care nurses as positive, comprehensive, and highly supportive.

*But in terms of the support, the explanations, the seriousness, and the professionalism…*

*[Caregiver]*

Many participants recognised the educational relationship with stoma care nurses as a fundamental step in accepting their new life circumstances.

*Encouraging me and saying, ‘Clean yourself up and change the pouch yourself,’ is a way of motivating you.*

*[Patient]*

*to lighten the situation*

*[Caregiver]*

*to help see something as normal*

*[Caregiver]*

Early therapeutic education provided from the time of stoma formation was perceived as instrumental in reducing potential challenges for both patients and caregivers

*P2: “Neither you nor I really know what we’re supposed to do, because especially under the skin barrier the pain gets worse, and we all know that, don’t we?”[Patient]*

*P4:“For me, it’s the itching”[Patient]*

*P2: “The itching, the pain […] I have pain too, and thank goodness you’re here [stoma care nurses] to tell us what we need to do”[Patient]*

*We didn’t have any real difficulties; in fact, we received a lot of help […] It was explained to us straight away. When he was in the hospital, the stoma nurse came, took us to the bathroom, and that’s where we had the first lesson.*

*[Caregiver]*

##### Peer Literacy

Peer exchanges emerged as an informal yet meaningful source of learning. Some participants reported sharing practical strategies, creative solutions, and personal experiences, highlighting how mutual support among patients and caregivers contributed to normalising challenges and strengthening confidence. Peer patient education emerged as a dimension that, although not formally structured within the care pathway, was clearly articulated by participants in the focus group and therefore represents a real and meaningful component worthy of recognition. Some participants reported sharing the creative solutions they had devised to overcome everyday challenges with the group.

*P2: “Has anyone with a stoma been to the beach?” [Patient]*

*P4: “I have. I went about three months after my surgery” [Patient]*

*P5: “I wanted to ask if there’s a pouch that’s suitable for going to the beach” [Patient]*

*In the end, we came up with some creative solutions ourselves*

*[Caregiver]*

*Because over time, you become empirical, you start trying to figure out what actually makes a difference*

*[Patient]*

In addition, some participants reported experiencing significant reassurance and practical benefit from the educational strategy implemented through focus group discussions.

*Creating a group of patients could be interesting, also because it helps you realise that some things are actually normal, for instance, when the pouch comes off. And then, of course, everyone finds their own solution, but even just knowing that…*

*[Caregiver]*

*I’ve been finding this very helpful. In groups, someone might say, ‘I went on a cruise, and this is how I dealt with it,’ or ‘When I go out for dinner, and something happens…’. Everyone brings their own experience, and it becomes a kind of listening group*

*[Caregiver]*

The dialogue reported below illustrates the powerful role of peer relationships. The conversational dynamic between the two patients enables the exploration of each other’s everyday experiences, fostering a process of identification through which participants project these experiences onto themselves and internalise what is shared.

*“Sometimes the stoma bothers me a little. I have been to the seaside. I do not practise physical exercise—actually, I never have—but I have many interests. I paint, I have written a book, I write poetry, and I have raised a family” [Patient]P5: “Have you been swimming?” [Patient]*

*P4: “* *Yes, I have done everything” [Patient]*

*P5: “Wearing a one-piece swimsuit?” [Patient]*

*P4: “* *Yes, a one-piece” [Patient]*

##### Relational Literacy

Several caregivers described developing a relational form of literacy as they negotiated their role within the triad of patient–caregiver–nurse. This learning process involved understanding responsibilities, seeking guidance, and observing how others managed daily care, shaping their sense of competence and involvement. Relational literacy emerges as caregivers learn to understand and negotiate their role not only through interaction with the stoma care specialist but also through engagement with other patients and their caregivers, drawing on their lived experiences. Most caregivers emphasised the importance of their supportive role in the care of the person living with a stoma.

*From my point of view, in terms of the help and support I provide, my main concern is what would happen if, for any reason, I couldn’t be there*

*[Caregiver]*

*If I couldn’t be there and he wasn’t able to manage on his own…*

*[Caregiver]*

Many caregivers actively posed numerous and specific questions, seeking information both from other caregivers and from patients living with a stoma.

*C3: But do you all need a caregiver, or has anyone managed to do it on their own? I mean… I can understand that the baseplate might be more complicated, but even changing the pouch—do you manage that by yourselves? [Caregiver]*

*P4: I manage everything on my own [Patient]*

*C1: He managed everything on his own […] After he was discharged from the hospital, he took care of everything himself. I later learned because someone else needed to know how to do it as well [Caregiver]*

*C3: Is this for the baseplate? [Caregiver]*

*P1: The hernia is what got me [Patient]*

*How do you do it? When you change it… Madam, since you do it on your own, do you do it standing up? And the baseplate—how do you manage that?*

*[Caregiver]*

*Do you change it in front of the mirror?*

*[Caregiver]*

#### 3.2.2. Time as a Protective and Regulatory Resource in Everyday Stoma Management

Time emerged as a crucial dimension in the experiences reported by all participants. Rather than being described as a purely chronological element, it appeared as a protective resource through which participants organised care practices, sought reassurance, and prepared themselves for social engagement.

##### Digital Time of Care

Remote communication with stoma care nurses was perceived as a valuable extension of care. Most participants described how digital contact, via phone call, email, or photos, provided timely reassurance and practical guidance, often preventing the need for in-person visits. Digital care time emerged as an aspect of considerable interest among participants. During the focus group, several examples were reported in which remote communication with the stoma care nurse allowed the issue to be resolved directly at the patient’s home, avoiding the need for an in-person assessment in the outpatient clinic.

*Personally, I found it very helpful. Being able to stay in contact—to write an email, but also to speak with you on the phone. Sometimes writing isn’t always easy.*

*[Caregiver]*

*Knowing that there is someone you can call when you find yourself in difficulty is very important. You can call, or even send a photo and ask, ‘What do you think about this?’ It’s not that we always have to come here—we can send an email, send a picture, and say, ‘Look, I’m in this situation, what do you think?’ Having a point of reference is really important for us.*

*[Caregiver]*

*Knowing that, whenever I need it, I can call […] and there is always advice on what to do*

*[Patient]*

##### Negotiating Before Social Engagement

A few participants highlighted the need for a preparatory mental space before engaging in social activities. A mental space of reassurance before social exposure, representing the time individuals need to feel safe before leaving home, travelling, going to the beach, or engaging in everyday activities such as shopping.

*I have to plan everything I need to do*

*[Patient]*

*I try not to travel in the morning […] because leaks can occur—sometimes the pouch is defective, and that can happen too.*

*[Patient]*

*We have to change the pouch after he eats, so if you go out for lunch you know that afterwards you’ll need to change it.*

*[Caregiver]*

*I’m Ukrainian, so my journey takes about two and a half days. When I have to travel there, I don’t eat for those two and a half days because I’m afraid—I mean, where would I manage all of this?*

*[Patient]*

*If we go out somewhere in the evening, he doesn’t have dinner. Once it happened on the motorway—the baseplate came off, and we had to stop. Since then, we’ve adopted this system: if we go somewhere, he eats at lunchtime but not in the evening, just some milk or tea, and then we can travel without worrying.*

*[Caregiver]*

#### 3.2.3. Living with a Stoma Requires Adaptation and Meaning-Making

This theme reflects how participants narrated their experience of living with a stoma, describing adaptation as an evolving process through which everyday practices, bodily changes, and personal meanings were gradually reinterpreted. Following stoma formation, the body undergoes a profound transformation, and individuals gradually learn to inhabit their “new” body. Before surgery, many bodily functions remain socially invisible and are taken for granted. The transformed body communicates meaning to others while simultaneously redefining the individual’s sense of self. In addition, all participants described an ongoing psychological appraisal of their new condition. While some perceived the stoma as an opportunity to continue living, others experienced it as a valuable resource that enabled them to regain control over their lives. For others, however, the stoma was perceived as an unavoidable path, with no alternative possibilities.

##### Embodied Communication in Everyday Life

Living with a stoma reshaped how participants experienced and presented their bodies in daily life. Clothing choice, social exposure, and bodily sensations acquired new meanings, reflecting the ongoing negotiation between visibility, comfort, and identity. Both patients and caregivers described multiple changes in everyday life compared with their lives before stoma formation. The transformation is not only physical but also embodied, influencing the way individuals experience and inhabit their bodies in the world. The transformed body communicates meaning to others while simultaneously reshaping the individual’s sense of self. The stoma becomes a form of social language, communicating meaning even in the absence of words.

*I go to the beach and wear a swimsuit. If people are curious, it’s usually children, and with them you can simply say it’s a dressing covering a wound. Adults might look, but it’s not really a problem*

*[Patient]*

Clothing may function as a form of protection, as people living with a stoma often express concern about keeping the appliance covered and not visible, particularly when wearing swimwear in social contexts.

*When I go to the beach wearing a swimsuit, this plate is visible, and it’s not very pleasant to look at*

*[Patient]*

*I won’t go to the beach until I’ve bought a suitable swimsuit*

*[Patient]*

Some participants described changes in sleep patterns, including reduced sleep due to the need to monitor the filling of the stoma bag, as well as improvements linked to fewer nocturnal awakenings previously caused by nocturia.

*There was this constant checking and touching to see how things were going. At night, especially in the early days, that was the main concern—feeling whether it needed to be changed.*

*[Patient]*

*At night I don’t sleep […] around midnight the evening really begins, and I stay up until three or four in the morning—watching television, using the computer, one thing or another. Then at about 4:30 I go to bed.*

*[Patient]*

*Actually, I’ll tell you, at night it’s even easier—I used to get up six or seven times a night.*

*[Patient]*

##### Resignation or a Coping Strategy for Survival?

Among oncology patients, the process of accepting the changes associated with stoma formation was described as variable both over time and in its expression; individuals oscillated between coping strategies that enabled continuity and moments of difficulty in adjusting to their new condition. For some participants, recognising that stoma formation represented the only viable option for survival became a starting point for acceptance.

*I’m still alive, you know. I’m an optimist.*

*[Patient]*

*I saw the surgery […] as something that saved my life.*

*[Patient]*

*Knowing that I could have something more controllable, because for me the stoma is more manageable, made it almost easier to accept.*

*[Patient]*

*The main things we used to do before, we still do.*

*[Patient]*

*I try to put these problems aside and focus on the things I used to do before. I’ve even managed to go to an exhibition and to the cinema, sometimes on my own, because I used to do that quite often.*

*[Patient]*

For others, this awareness was instead characterised by a sense of resignation.

*It felt like a stab*

*[Patient]*

*I had no choice*

*[Patient]*

*So basically, from one day to the next, we suddenly found ourselves facing a problem that escalated from zero to a hundred.*

*[Caregiver]*

*I lost three years of my working life.*

*[Caregiver]*

## 4. Discussion

A deeper understanding of the perceptions and lived experiences of individuals with enterostomies or urostomies, as well as their caregivers, is crucial for tailoring educational content and care interventions to their specific needs. The findings of this study shed light on how adult cancer patients with entero-urostomies and their caregivers experience and make sense of PE activities.

Our findings suggest that the interactions generated through the focus group methodology enabled patients and caregivers to engage with the group as a community, allowing them to raise questions and reflect on the responses received in relation to their own everyday lives.

### 4.1. Translational Patient Education as a Co-Constructed Educational Process

The main construct emerging from this study can be summarised as a complementary dynamic between knowledge and experience. The stoma care nurse provides professional guidance on what can be done in stoma management; patients, through their lived experience, demonstrate how it can be done in everyday life; and caregivers, learning from both professionals and patients, engage with both dimensions of what and how.

Previous literature has described peer support, experiential knowledge and patient–professional partnerships [31,32,33] without recognising their integration within a single, dynamic educational process. Our findings build on these perspectives by suggesting that, in the context of stoma care, these elements do not operate independently but are continuously integrated through a multidirectional educational process mediated by the stoma care nurse. We offer a conceptualisation that brings these existing dimensions together into a coherent educational process.

As it enables the analysis and integration of three distinct forms of literacy (clinical/specialist, peer, and relational) within the context of therapeutic education, we propose translational patient education as an educational ecosystem in which the stoma care nurse not only provides therapeutic education but also facilitates and mediates the multidirectional exchange of knowledge between patients and caregivers. Within this framework, patients and caregivers are recognised as active contributors who transform lived experience into transferable knowledge, resulting in a continuous process of knowledge circulation and co-construction.

**Figure 1:**
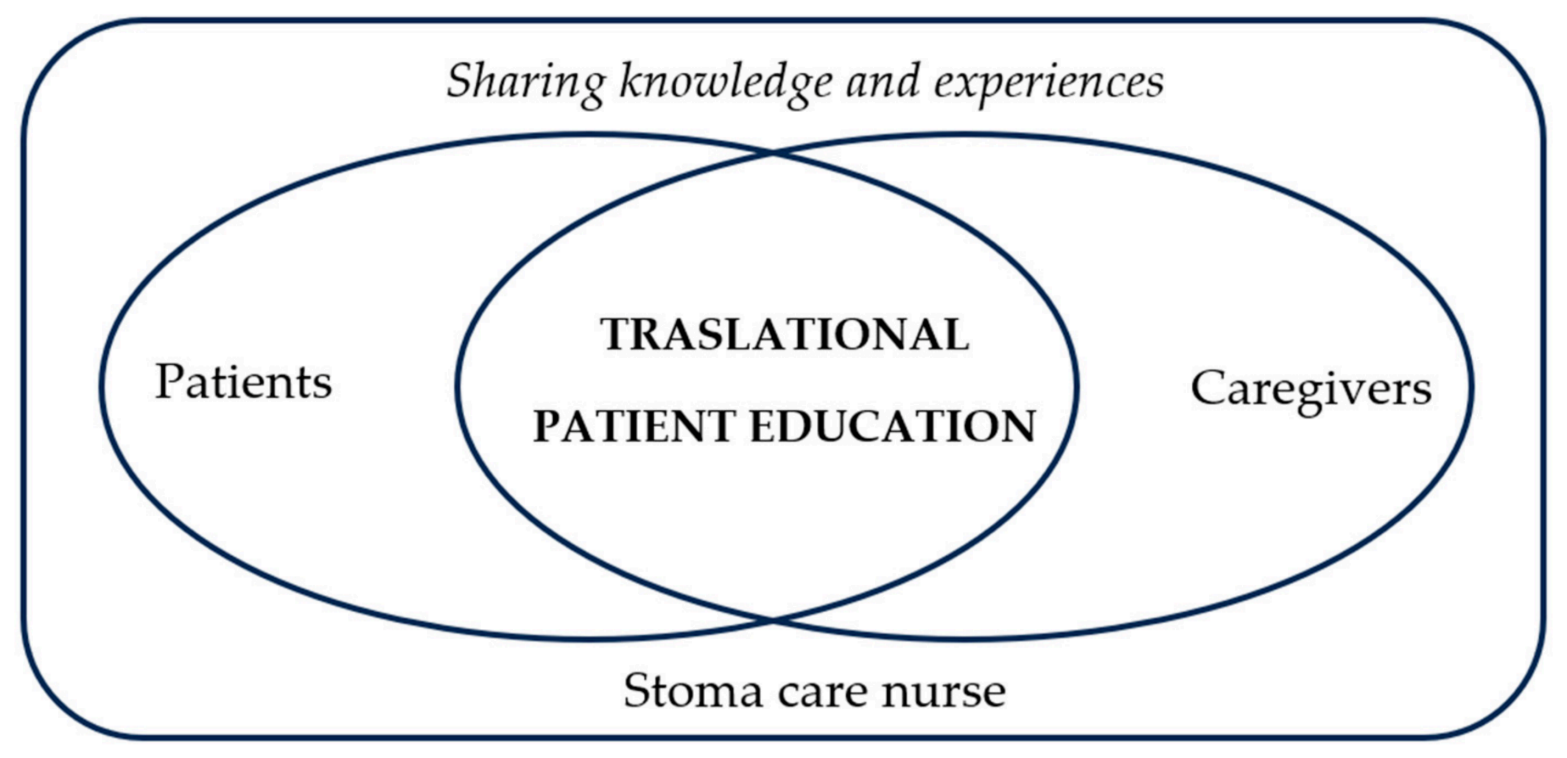
Translational patient education as a co-constructed educational process.

This finding shifts the educational focus beyond the content and delivery of educational interventions, drawing attention to the value of the interactions and exchanges that emerge within peer groups in the presence of caregivers. In this context, a dialogical space is created in which two peer subgroups—patients and caregivers—interact with each other, with their interactions mediated and catalysed by the stoma care nurse, fostering the collective generation of experiential knowledge. Figure 1 provides a visual representation of the concept of translational patient education.

### 4.2. Time as a Protective and Regulatory Resource in Everyday Stoma Management

Both patients and caregivers perceived time as a space of safety within which they could progressively learn how to manage everyday life with the stoma. In this context, the sense of closeness provided by telenursing, even in the absence of physical presence, was perceived as highly valuable and positively received. Among oncology patients, the perception of the home as a ‘safe place’ for care delivery through telenursing aligns with the existing body of literature [34,35,36].

Negotiation prior to social exposure enables patients and caregivers to navigate everyday life. The planning of daily activities functions as a safety strategy, addressing the perceived risks associated with possible stoma-related complications [37]. The conception of time as a space of safety may be linked to the concept of anticipatory coping. This construct, applicable to healthcare and the management of chronic illness, explains how patients and caregivers anticipate potential problems and organise their everyday lives in order to prevent them [38,39,40,41]. This negotiation is relational in nature, involving both the patient and the caregiver, who collaboratively engage in a process of regulating the potential risks associated with living with a stoma.

### 4.3. Living with a Stoma Requires Adaptation and Meaning-Making

As widely reported in the literature, stoma formation represents not only a surgical intervention but also a profound embodied transformation [42,43]. Participants described a gradual process of learning to inhabit a ‘new’ body, in which previously invisible bodily functions become visible and socially meaningful. The process of psychological appraisal and acceptance appeared heterogeneous among oncology patients: while some interpreted the stoma as an opportunity to continue living or regain control over their lives, others perceived it as an unavoidable pathway linked to survival. These findings suggest that adjustment to life with a stoma involves complex embodied, relational, and meaning-making processes that unfold over time.

### 4.4. Limitations, Strengths, and Future Perspectives

Although the authors recognise the value of this focus group in enabling an in-depth exploration of the perceptions and experiences of patients with enterostomies or urostomies and their caregivers, several limitations should be acknowledged. First, caution is warranted in generalising the findings, and further studies with more age-diverse samples are recommended. Indeed, although the sample size was adequate per the methodology, it consisted predominantly of patients and caregivers aged ≥65 years. This may have influenced the variability of responses, particularly regarding intimate and personal aspects. Furthermore, although the focus group was conducted by a nurse experienced in this methodology and trained in PE, the possibility of social desirability bias cannot be ruled out, as participants may have provided responses they perceived as more acceptable or aligned with the study’s or group’s expectations.

Undoubtedly, the principal strength of this study is the proposal of the construct of translational patient education, which, to our knowledge, has not yet been explored in the literature. The innovative findings of the present study open avenues for further research. A deeper understanding of the dynamic and circular interaction between patients, caregivers, and the stoma care nurse requires additional investigation. The development of a theoretical framework could support the tailoring of educational programmes, enabling the identification of appropriate modalities and timing for the delivery of translational patient education interventions.

## 5. Conclusions

This study highlights the value of PE as a dynamic and relational process that extends beyond the transmission of information, encompassing the interaction between patients, caregivers, and healthcare professionals. Further advancing the understanding developed in our previous study [44], these results stress the importance of shared experiences, peer interaction, and professional guidance in fostering self-care, meaning-making, and emotional adjustment. The proposed perspective of translational patient education reframes patient education as a multidirectional process in which clinical, experiential, and relational knowledge are continuously exchanged through the mediating role of the stoma care nurse. Additionally, the perception of time and care contexts as ‘spaces of safety’, alongside the role of telenursing, emphasises the need for flexible and person-centred approaches. Despite the study’s limitations, these results provide a valuable foundation for the development of tailored educational interventions and call for further research to validate and expand this emerging conceptual model across diverse populations and settings.

## Figures and Tables

**Table 1:** Participants’ sociodemographic and clinical characteristics (N = 28).

	Patients N (%)	Caregivers N (%)
**Total**	15 (100)	13 (100)
**Age (years)**		
mean ± SD (range)	70.5 ± 5.9 (60–82)	68.3 ± 2.8 (62–74)
≥65 years	11 (73.3)	11 (84.6)
<65 years	2 (13.3)	1 (7.7)
Undeclared	2 (13.3)	1 (7.7)
**Gender**		
Male	9 (60.0)	4 (30.8)
Female	4 (40)	9 (69.2)
**Education**		
Primary	0	1 (7.7)
Lower secondary	2 (13.3)	3 (23.1)
Upper secondary	7 (46.7)	6 (46.1)
Bachelor’s degree	1 (6.7)	2 (15.4)
Master’s degree	4 (26.7)	1 (7.7)
Undeclared	1 (6.7)	0
**Marital status**		
Single	1 (6.7)	0
Married/Cohabiting	12 (80)	12 (92.3)
Divorced/Separated	1 (6.7)	1 (7.7)
Widowed	0	0
Undeclared	1 (6.7)	0
**Currently employed**		
Yes	1 (6.7)	0
No (Retired)	13 (86.7)	12 (92.3)
Undeclared	1 (6.7)	1 (7.7)
**Lives alone**		
Yes	0	1 (7.7)
No	10 (66.7)	8 (61.5)
Undeclared	5 (33.3)	4 (30.8)
**Children**		
Yes	13 (86.7)	10 (76.9)
No	1 (6.7)	2 (15.4)
Undeclared	1 (6.7)	1 (7.7)
**Duration of stoma**		
2020	2 (13.3)	/
2021	0	/
2022	3 (20)	/
2023	5 (33.3)	/
2024	2 (13.3)	/
2025	3 (20)	/
**Type of stoma**		
Colostomy	7 (46.7)	/
Ileostomy	4 (26.7)	
Urostomy (ileal conduit)	3 (20)	/
Colostomy and Nephrostomy	1 (6.7)	/
**Stoma permanence**		
Temporary	4 (26.6)	/
Permanent	11 (73.3)	/
**Chemotherapy**		
Yes	8 (53.3)	/
No	2 (13.3)	/
Undeclared	5 (33.3)	/
**Radiotherapy**		
Yes	4 (26.7)	/
No	3 (20)	/
Undeclared	8 (53.3)	/

**Table 2:** Identified qualitative themes and subthemes.

Theme	Subtheme
Translational Patient Education as a Co-constructed Educational Process	Ostomy Specialist Literacy Peer literacy Relational literacy
Time as a Protective and Regulatory Resource in Everyday Stoma Management	Digital time of care Negotiating before social engagement
Living with a stoma requires adaptation and meaning-making	Embodied communication in everyday life Resignation or a coping strategy for survival?

## Data Availability

The datasets generated and/or analysed during the current study are not publicly available but are available from the corresponding author on reasonable request due to privacy restrictions and ethical issues.

## Supplementary Materials

The following supporting information can be downloaded at: https://www.mdpi.com/article/10.3390/healthcare14162501/s1, Table S1: COREQ (COnsolidated criteria for REporting Qualitative research) Checklist.
